# Comparative Study of Pulmonary Combined Large-Cell Neuroendocrine Carcinoma and Combined Small-Cell Carcinoma in Surgically Resected High-Grade Neuroendocrine Tumors of the Lung

**DOI:** 10.3389/fonc.2021.714549

**Published:** 2021-09-22

**Authors:** Yanan Wang, Fangfei Qian, Ya Chen, Zhengyu Yang, Minjuan Hu, Jun Lu, Yanwei Zhang, Wei Zhang, Lei Cheng, Baohui Han

**Affiliations:** Department of Pulmonary, Shanghai Chest Hospital, Shanghai Jiao Tong University, Shanghai, China

**Keywords:** pulmonary neuroendocrine carcinoma, pulmonary combined large-cell neuroendocrine carcinoma, combined small-cell lung cancer, prognosis, propensity score matching

## Abstract

**Objectives:**

Pulmonary large-cell neuroendocrine carcinoma (LCNEC) and small-cell lung cancer (SCLC) are both classified as pure and combined subtypes. Due to the low incidence and difficult diagnosis of combined LCNEC (C-LCNEC) and combined SCLC (C-SCLC), few studies have compared their clinical features and prognosis.

**Materials and Methods:**

We compared the clinical features, mutation status of driver genes (*EGFR, ALK, ROS1, KRAS*, and *BRAF*), and prognosis between C-LCNEC and C-SCLC. Univariate and multivariate Cox regression analyses were applied for survival analysis.

**Results:**

We included a total of 116 patients with C-LCNEC and 76 patients with C-SCLC in the present study. There were significant differences in distribution of smoking history, tumor location, pT stage, pN stage, pTNM stage, visceral pleural invasion (VPI), and combined components between C-LCNEC and C-SCLC (*P<*0.05 for all). C-SCLC was more advanced at diagnosis as compared to C-LCNEC. The incidence of *EGFR* mutations in C-LCNEC patients was higher than C-SCLC patients (25.7 *vs.* 5%, *P*=0.004). We found that tumor size, pN stage, peripheral CEA level, and adjuvant chemotherapy were independently prognostic factors for DFS and OS in C-LCNEC patients, while peripheral NSE level, pT stage, pN stage, VPI and adjuvant chemotherapy were independently associated with DFS and OS for C-SCLC patients (*P<*0.05 for all). Propensity score matching with adjustment for the confounders confirmed a more favorable DFS (*P*=0.032) and OS (*P*=0.019) in patients with C-LCNEC in comparison with C-SCLC patients upon survival analysis.

**Conclusions:**

The mutation landscape of driver genes seemed to act in different way between C-SCLC and C-LCNEC, likely by which result in clinical phenotype difference as well as better outcome in C-LCNEC.

## Introduction

Neuroendocrine carcinoma of the lung, which accounts for about 15–20% of primary lung cancer, is divided into four categories: small-cell lung cancer (SCLC), typical carcinoid, atypical carcinoid, and large-cell neuroendocrine carcinoma (LCNEC) (1, 2). Pulmonary large-cell and small-cell neuroendocrine carcinomas are classified as high-grade neuroendocrine carcinomas (HGNEC), characterized by poor histologic differentiation, high aggressiveness, and poor prognosis (3). In 2015, the World Health Organization (WHO) classification divided SCLC into pure SCLC and combined SCLC (C-SCLC). C-SCLC is defined as a SCLC type that is mixed with other components of non-small-cell lung cancer (NSCLC), such as adenocarcinoma (AD), squamous cell carcinoma (SCC), large-cell carcinoma (LCC), LCNEC, and so on. C-SCLC accounts for 2 to 28% of all SCLC (4–6). Like SCLC, LCNEC is also divided into pure LCNEC and combined LCNEC (C-LCNEC), and C-LCNEC is the LCNEC that combined with AD, SCC, and other rare types such as spindle-cell carcinoma and giant-cell carcinoma. The reported incidence of C-LCNEC ranged from 10 to 49% (7–9). Current studies on LCNEC and SCLC generally take all components as a whole instead of specified analysis with focus on pure and combined parts (10–12). Owing to the rarity and the difficulty in diagnosis of C-LCNEC and C-SCLC, it is lack of the statistical description of their clinical features, genomic landscape, prognosis, and the relevant comparisons. The diagnosis of pulmonary neuroendocrine tumors has been advanced in recent years by the development of pathological technology and the wide application of surgery in multimodal treatment of lung cancer (13, 14). Therefore, genomic and clinical analysis on specific subtypes will take the advantage of increased diagnosis to improve the treatment guidance for SCLC and LCNEC. To our knowledge, no large sample studies up to now have compared C-LCNEC with C-SCLC in terms of clinical features and genomic mutation landscape. We conducted this study to fill this gap and wish the results may provide new insights into the treatment of resected C-LCNEC and C-SCLC.

## Material and Methods

### Patients

We retrospectively analyzed 1,250 patients with high-grade neuroendocrine carcinoma who underwent pulmonary resection in our organization. All surgically resected specimens were independently reviewed by two professional pathologists according to the 2015 WHO criteria for pulmonary neuroendocrine carcinoma (1). The initial diagnosis and classification of the C-LCNEC and C-SCLC were based on the neuroendocrine tumor morphology, which was further confirmed by immunohistochemistry. The tumor staging was based on the 8th edition TNM staging system proposed by the International Association for the Study of Lung Cancer (IASLC) (15). The treatment response was evaluated according to the Response Evaluation Criteria in Solid Tumors (RECIST) version 1.1. Besides NSCLC components, LCNEC might also be mixed with SCLC, but these tumors are classified as C-SCLC and will be excluded from our study. Other exclusion reasons included uncertain diagnosis, palliative surgery, and incomplete medical record and follow-up data.

**Figure 1:**
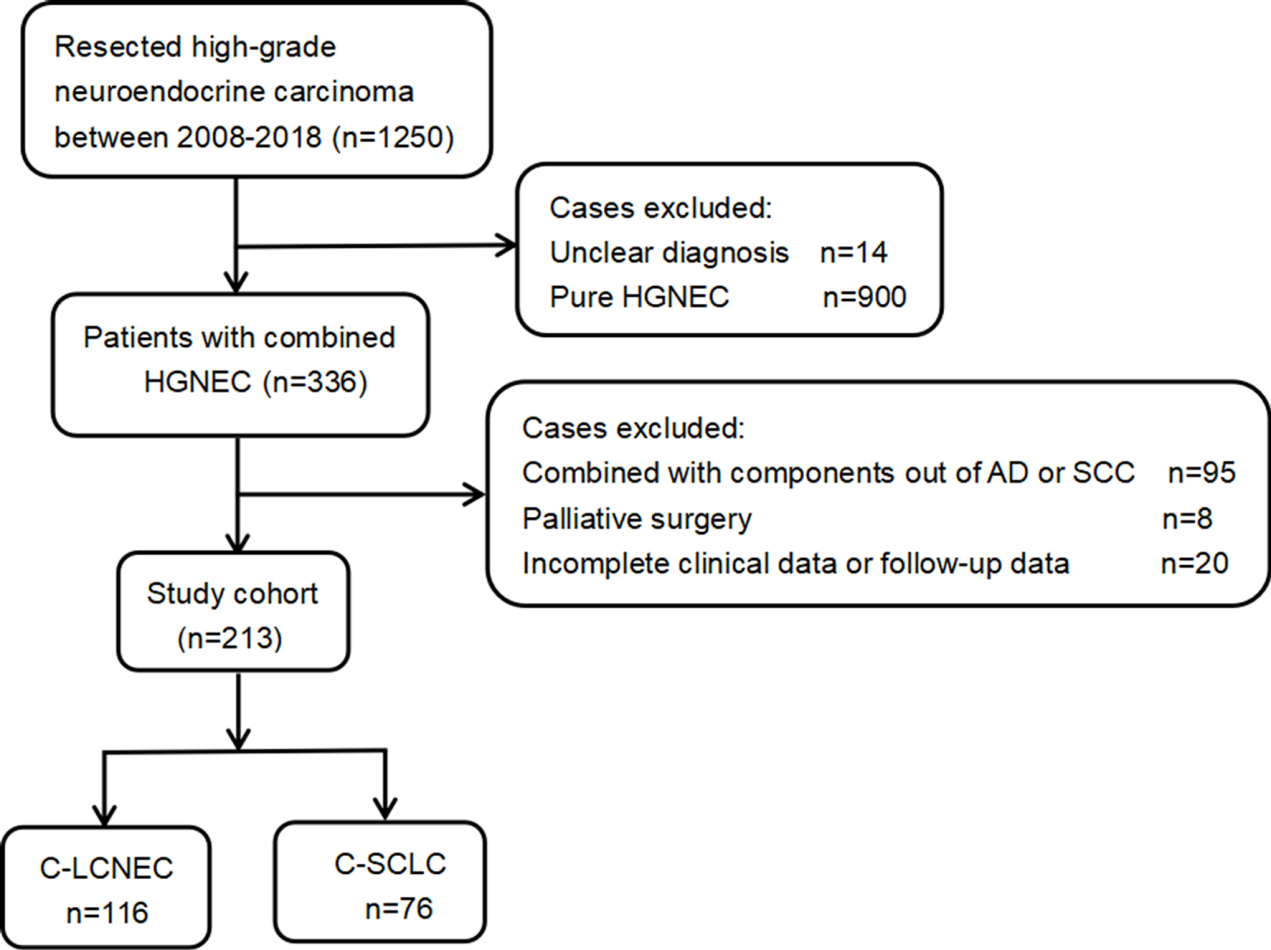
The workflow of patient inclusion for the present study.

As presented by the study flow chart (**Figure 1**), we finally included 192 cases of surgically resected combined high-grade neuroendocrine carcinoma in total. Specially, there were 116 patients with C-LCNEC and 76 patients with C-SCLC. The studies involving human participants were reviewed and approved by Shanghai Chest Hospital and affiliation of ethics committee. A written informed consent was signed by included patients before they donated tumor tissues for the purpose of scientific research.

### Molecular Analysis

The patient’s genomic DNA and RNA were extracted from formalin-fixed, paraffin-embedded tumor tissues and were subjected to the analysis of genetic alterations. We evaluated *EGFR* mutations by amplification refractory mutation system (ARMS) method, *ALK* expression by immunohistochemistry (IHC), ROS1 fusion by *in situ* hybridization (FISH), and *KRAS/BRAF* mutations by polymerase chain reaction (PCR) amplification method.

### Follow-Up

Patients were diagnosed between 2008 and 2018, with median follow-up of 76 months and 55.7% deaths at the end of follow-up. Survival information was obtained through inpatient and outpatient records or telephone. Routine examinations such as chest computed tomography (CT) scans, brain magnetic resonance imaging or CT, tumor markers, and the neck and abdominal ultrasound were performed every 3 months for the first 2 years after surgery, followed by every 6 months examinations until 2–5 years. After 5 years, the patients were examined once a year. Patients were censored who were lost to follow-up or did not achieve the endpoint event. Disease-free survival (DFS) was defined as the time from surgery to disease recurrence or last follow-up if censored. Overall survival (OS) was the period between the date of surgery and death, but was extended to the end of follow-up in the presence of censored situation.

### Statistical Analysis

We compared the differences in clinicopathological characteristics between C-LCNEC and C-SCLC patients. Chi-square test or Fisher’s exact test was used for the comparisons in presence of discrete variables. As a non-parametric analysis method, Mann-Whitney U test was adopted for comparisons of continuous variables that were distributed non-normally. Student t test was used for normal distribution variables. Survival analysis was performed by Cox proportional hazards regression model, and the survival difference was visualized by Kaplan–Meier curve and log-rank test.

To reduce the selection bias as much as possible, propensity score matching (PSM) method was applied to sample C-LCNEC and C-SCLC patients at a 1:1 ratio *via* nearest-neighbor method without replacement (16). Propensity scores for included patients were calculated by a multiple logistic regression with adjustment for clinical features including gender, age, smoking history, primary site, laterality, tumor location, tumor size, pT stage, pN stage, VPI, combined components, CEA, CYFRA21-1, SCCA, NSE, adjuvant chemotherapy, and postoperative adjuvant radiotherapy (PORT). Finally, A total of 75 pairs were successfully matched. We used the SPSS software of version 26.0 (IBM Corporation, Armonk, NY, USA) for statistical analysis, and two-tailed *P*<0.05 was considered statistically significant.

## Result

### Patient Population

The patients’ clinicopathologic characteristics are shown in **Table 1**. We included 116 patients pathologically diagnosed as C-LCNEC, and other 76 patients with C-SCLC. C-SCLC patients had a higher proportion of smoking than C-LCNEC patients (*P*=0.027) and tended to be central-type carcinoma (*P*=0.015). The pathological staging of C-SCLC was more advanced compared to C-LCNEC, in terms of pT, pN, and pTNM stage (*P*=0.012, 0.020, and 0.003; respectively). C-LCNEC appeared to have a higher incidence of VPI than C-SCLC (*P*=0.002). Among 116 C-LCNEC patients, the most common were LCNEC combined with adenocarcinoma (LCNEC/AD, 70.7%, n=82), and then LCNEC combined with squamous cell carcinoma (LCNEC/SCC, 29.3%, n=34). As for C-SCLC, the percentage of SCLC plus adenocarcinoma and squamous cell carcinoma was 50%, respectively. The proportion of combined with AD in C-LCNEC was higher than that in C-SCLC (P=0.013).To adjust the confounding factors between the two groups, we sampled the two patient population with the PSM method. We found similar results upon PSM adjustment by such comparisons mentioned above (**Table 1**).

**Table 1:** Clinical demographics before and after propensity score matching for patients with resected C-LCNEC and C-SCLC.

Characteristic	Before propensity score matching			After propensity score matching		
	C-LCNEC (n = 116) (%)	C-SCLC (n = 76) (%)	*P*	C-LCNEC (n = 75) (%)	C-SCLC (n = 75) (%)	P
**Gender**			0.117			0.440
** Male**	96(82.8)	69(90.8)		65 (86.7)	68 (90.7)	
** Female**	20(17.2)	7(9.2)		10 (13.3)	7 (9.3)	
**Age(y)**			0.872			0.869
** <65**	67(57.8)	43(56.6)		42 (56.0)	43 (57.3)	
** ≥65**	49(42.2)	33(43.4)		33 (44.0)	32 (42.7)	
**Smoking History**			**0.027**			0.299
** Yes**	64(55.2)	54(71.1)		47 (62.7)	53 (70.7	
** No**	52(44.8)	22(28.9)		28 (37.3)	43 (29.3)	
**Primary Site**			0.374			0.227
** Upper lobe**	69(59.5)	45(59.2)		41 (54.7)	44 (58.7)	
** Middle lobe**	4(3.4)	6(7.9)		2 (2.7)	6 (8.0)	
** Lower lobe**	43 (37.1)	25 (32.9)		32 (42.7)	25 (33.3)	
**Laterality**			0.603			0.511
** Left**	49 (42.2)	35 (46.1)		31 (41.3)	35 (46.7)	
** Right**	67 (57.8)	41 (53.9)		44 (58.7)	40 (53.3)	
**Tumor location**			**0.015**			0.157
** Central**	22(19.0)	26(34.2)		19 (25.3)	27 (36.0)	
** Peripheral**	94(81.0)	49(64.5)		56 (74.7)	48 (64.0)	
**Tumor size, cm (Mean ± SD)**	3.55 ± 1.62	3.73 ± 1.96	0.186	4.30 ± 2.3	4.26 ± 2.2	0.221
**pT stage**			**0.012**			0.265
** T1**	25(21.6)	28(36.8)		22 (29.3)	28 (37.3)	
** T2**	71(61.2)	28(36.8)		39 (52.0)	27 (36.0)	
** T3**	16(13.8)	16(21.1)		11 (14.7)	16 (4.0)	
** T4**	4(3.4)	4(5.3)		3 (4.0)	4 (5.3)	
**pN stage**			**0.020**			0.495
** N0**	68(58.6)	29(38.2)		24 (44.0)	29 (38.7)	
** N1**	17(14.7)	15(19.7)		17 (22.7)	14 (18.7)	
** N2**	31(26.7)	32(42.1)		25 (33.3)	32 (42.7)	
**pTNM stage**			**0.003**			0.138
**I**	51 (44.0)	19 (25.0)		24 (32.0)	19 (25.3)	
**II**	31 (26.7)	17 (22.4)		23 (30.7)	16 (21.3)	
**III**	34 (29.3)	40 (52.6)		28 (37.3)	40 (53.3)	
**VPI**			**0.002**			0.611
**With**	64(55.2)	25(32.9)		46 (61.3)	49 (65.3)	
**Without**	52(44.8)	51(67.1)		29 (38.7)	26 (34.7)	
**Combined components**			**0.013**			0.189
** AD**	82 (70.7)	38 (50.0)		45 (60.0)	37 (49.3)	
** SCC**	34 (29.3)	38 (50.0)		30 (40.0)	38 (50.7)	
**CEA, ng/ml**			0.231			0.244
**≤5**	65(56.0)	42(55.3)		43 (57.3)	42 (56.0)	
**>5**	40 (34.5)	17(22.4)		27 (36.0)	17 (22.7)	
** Unknown**	11(9.5)	17 (22.4)		0	0	
**CYFRA21-1, ng/ml**			0.739			0.810
**≤5**	85 (73.3)	49 (64.5)		57 (76.0)	49 (65.3)	
**>5**	20 (17.2)	10 (13.2)		13 (17.3)	10 (13.3)	
** Unknown**	11(9.5)	17(22.4)		5 (6.7)	16 (21.3)	
**SCCA, ng/ml**			0.190			0.346
**≤1.5**	82(70.7)	51 (67.1)		52 (69.3)	51 (68.0)	
**>1.5**	23 (19.8)	8 (10.5)		18 (24.0)	8 (10.7)	
** Unknown**	11(9.5)	17 (22.4)		5 (6.7)	16 (21.3)	
**NSE, ng/ml**			0.552			0.975
**≤16**	91 (78.4)	53 (69.7)		63 (84.0)	53 (70.7)	
**>16**	14 (12.1)	6 (7.9)		7 (9.3)	6 (8.0)	
** Unknown**	11 (9.5)	17 (22.4)		5 (6.7)	16 (21.3)	
**CA125, kU/L**			0.546			0.365
**≤35**	95 (81.9)	55 (72.4)		62 (82.7)	55 (73.3)	
** >35**	10 (8.6)	4 (5.3)		8 (10.7)	4 (5.3)	
** Unknown**	11 (9.5)	17 (22.4)		5 (6.7)	16 (21.3)	
**Adjuvant chemotherapy**			0.546			0.852
**Yes**	88 (75.9)	55 (72.4)		60 (80.0)	55 (73.3)	
** NSCLC-regimen**	51 (58.0)	17 (30.9)		26 (43.4)	17 (30.9)	
** SCLC-regimen**	37 (42.0)	38 (69.1)		34 (56.3)	38 (69.1)	
**No**	28 (24.1)	21 (27.6)		15 (20.0)	20 (26.7)	
**PORT**			0.599			0.334
** Yes**	18 (15.5)	20 (26.3)		56 (74.7)	55 (73.3)	
** No**	98 (84.5)	56 (73.7)		19 (25.3)	20 (26.7)	

C-LCNEC, combined large-cell neuroendocrine carcinoma; C-SCLC, combined small-cell lung cancer; pTNM stage, pathological tumor node metastasis staging; VPI, Visceral pleural invasion; AD, adenocarcinoma; SCC, squamous cell carcinoma; CEA, carcinoembryonic antigen; CYFRA21-1, Cytokeratin-19-fragment; SCCA, squamous cell carcinoma antigen; NSE, neuron-specific enolase; PORT, postoperative adjuvant radiotherapy.

In bold: P < 0.05.

### Genetic Alterations

**Figure 2:**
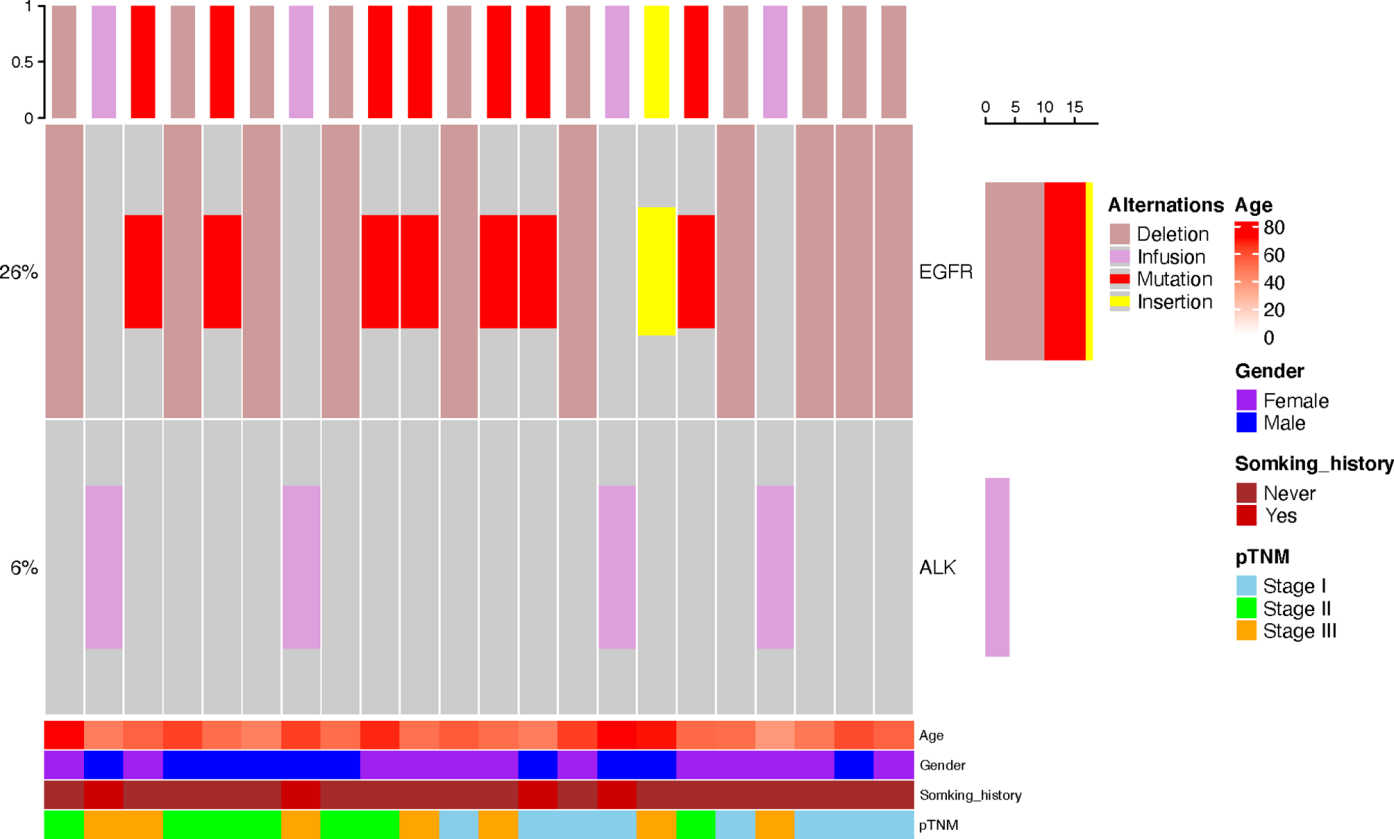
Genetic alternations of patients with resected C-LCNEC and C-SCLC.

The mutation data of driver genes such as *EGFR*, *ALK*, *ROS1*, *KRAS*, and *BRAF* were available in 110 patients, including 70 C-LCNEC and 40 C-SCLC patients. As shown in **Figure 2**, we only found the alterations of *ALK* and *EGFR* genes in these patients. The incidence of *EGFR* mutations in C-LCNEC patients was found to be higher than C-SCLC patients (25.7 *vs.* 5%, *P*=0.004). Specially, there were 18 patients with *EGFR* mutations of C-LCNEC, including 10 patients with 19 exon deletions, 7 patients with 21 exon *L858R* mutation, and 1 patient with exon 20 insertion. For C-SCLC patients, we only identified two patients with *EGFR* 19 exon deletions. The incidence of *ALK* rearrangement were relatively lower, with only four patients detected in C-LCNEC, but none were detected in C-SCLC patients, with insignificant statistical differences (5% *vs.* 0, *P*=0.102) likely due to limited sample size.

### Postoperative Treatment Modalities

A total of 143 patients received adjuvant chemotherapy, including 88 patients with C-LCNEC and 55 patients with C-SCLC. Adjuvant chemotherapy regimens were divided into two types: SCLC regimens (etoposide plus cisplatin or carboplatin, EP/EC) and NSCLC regimens (platinum-based combined pemetrexed, gemcitabine, docetaxel, paclitaxel, or vinorelbine). Of the 88 C-LCNEC patients, 51 patients received NSCLC regimens, of which pemetrexed/cisplatin or carboplatin contained 32 (62.7%), gemcitabine/docetaxel/paclitaxel/vinorelbine–platinum contained 19 (37.3%), and the remaining 37 patients received SCLC regimens, including 24 patients with EC regimen and 13 patients with EP regimen. Among the 55 C-SCLC patients, 17 patients received NSCLC regimens, including 10 patients receiving pemetrexed/cisplatin or carboplatin regimen and 7 patients receiving gemcitabine/docetaxel/paclitaxel/vinorelbine–platinum regimen, and 38 patients received the SCLC regimens, of which 22 were EC regimen and 16 were EP regimen. As shown in **Supplemental Table 1**, a total of nine patients with C-LCNEC who developed distant metastases after surgery were treated with tyrosine Kinase Inhibitor (TKI). Among them, four patients harboring EML4-ALK received crizotinib, and five patients with EGFR 19del/L858R mutations received either first-generation TKI (gefitinib, icotinib, or erlotinib) or second-generation TKI (afatinib). Only one patient with C-SCLC harboring EGFR 19del received icotinib treatment. The overall ORR (the proportion of patients with a confirmed complete or partial response) rate was 60%.

### Survival Comparison Between C-LCNEC and C-SCLC

**Figure 3:**
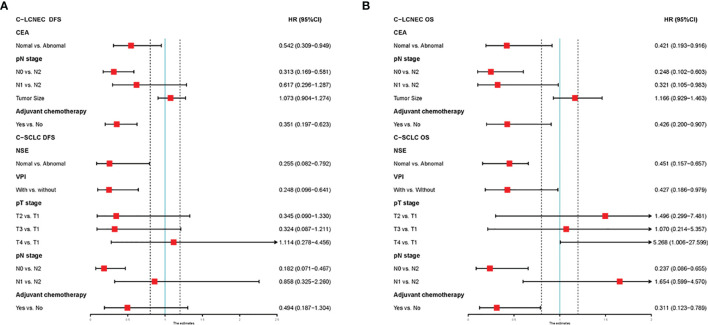
Multivariate Cox regression analysis on clinical characteristics for DFS **(A)** and OS **(B)**.

**Figure 4:**
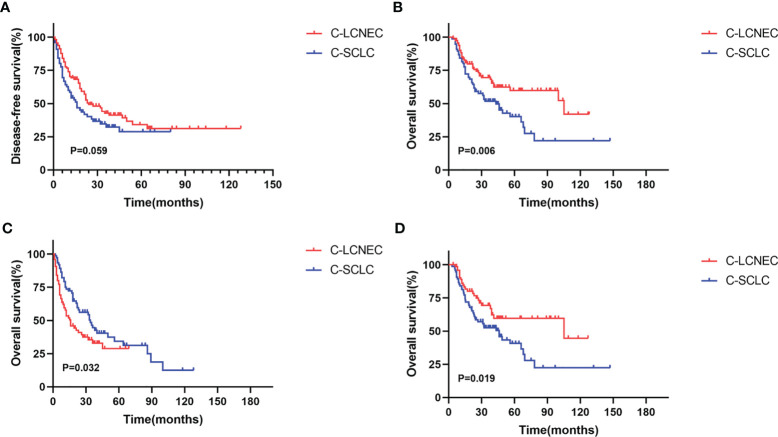
Kaplan-Meier curves for Disease-free survival **(A, C)** and overall survival **(B, D)** before matching and after matching between C-LCNEC and C-SCLC.

We enrolled the factors that might affect DFS and OS in C-LCNEC and C-SCLC patients into Cox regression models for survival analysis (**Tables 2**, ** 3**). We initially made univariate Cox analysis and found tumor size, pN stage, CEA, and adjuvant chemotherapy were the main DFS modulator for C-LCNEC with statistical significance (*P*<0.05 for all, **Table 2**). On the other hand, tumor size, pT stage, pN stage, tumor location, peripheral VPI and NSE levels, and adjuvant chemotherapy were significantly associated with DFS in C-SCLC by univariate survival analysis (*P*<0.05 for all, **Table 2**). For OS, univariate analysis indicated that age, tumor size, pT stage, pN stage, CEA, and adjuvant chemotherapy were main predictors for C-LCNEC patients’ survival (*P*<0.05 for all; respectively, **Table 3**). On the other hand, pT stage, pN stage, peripheral VPI and NSE levels, and adjuvant chemotherapy were survival predictors in C-SCLC patients as indicated by univariate modeling (*P*<0.05 for all, **Table 3**). These main survival effectors in univariate Cox regression models were further included in multivariate analysis. As results, we found that tumor size, pN stage, peripheral CEA level, and adjuvant chemotherapy were independently associated with DFS and OS in C-LCNEC patients (**Figure 3**). For C-SCLC patients, NSE, pT stage, pN stage, VPI, and adjuvant chemotherapy were independently predictors for DFS and OS (**Figure 3**). We next compared the outcomes of patients between C-LCNEC and C-SCLC and found that there was a longer OS in patients with C-LCNEC (*P*=0.006, **Figure 4B**). But we only observed an insignificant trend towards favorable DFS in patients with C-LCNEC (*P*=0.059, **Figure 4A**). PSM method adopted to reduce the confounders between the groups resulted in better DFS and OS of C-LCNEC patients than C-SCLC patients, highlighting the indeed difference in prognosis between the two lung cancer subtypes (for DFS, *P*=0.032, and for OS, *P*=0.019; **Figures 4C, D**).

**Table 2:** Impact of clinical characteristics on disease-free survival in patients with resected C-LCNEC and C-SCLC by univariate Cox analysis.

Characteristic	C-LCNEC			C-SCLC			
	HR	95% Cl	P	HR	95% Cl	P	
Age ≥65 (***vs.***<65)	1.018	0.618–1.675	0.944	0.652	0.363–1.173	0.154	
Gender male (***vs.*** Female)	1.134	0.592–2.172	0.705	0.921	0.330–2.569	0.875	
Smoking history (***vs.*** No)	1.019	0.623–1.667	0.946	0.834	0.433–1.605	0.587	
Tumor Size	1.186	1.007–1.396	**0.041**	1.216	1.057–1.397	**0.006**	
pT stage (***vs.*** T1)							
T2	0.97	0.517–1.823	0.926	1.415	0.686–2.917	0.347	
T3	1.95	0.887–4.283	0.096	3.053	1.42–6.562	**0.004**	
T4	2.656	0.582–12.129	0.207	4.151	1.341–12.849	**0.014**	
pN stage (***vs.*** N2)							
N0	0.266	0.154–0.459	**<0.001**	0.265	0.126–0.557	**<0.001**	
N1	0.604	0.305–1.195	0.148	0.962	0.490–1.886	0.909	
Tumor location Central (***vs.*** Peripheral)	1.340	0.683–2.630	0.395	1.959	1.081–3.551	**0.027**	
Combined components AD (***vs.*** SCC)	0.830	0.472–1.459	0.517	0.743	0.420–1.318	0.309	
VPI (***vs.*** Without)	1.014	0.621–1.655	0.955	2.742	1.506–4.995	**0.001**	
Primary Site (***vs.*** upper lobe)							
Middle lobe	0.657	0.396–1.091	0.105	0.901	0.485–1.673	0.742	
Lower lobe	1.805	0.629–5.179	0.272	1.546	0.564–4.238	0.398	
CEA normal (***vs.*** Abnormal)	0.454	0.268–0.771	**0.003**	1.798	0.900–3.592	0.096	
CYFRA21-1 normal (***vs.*** Abnormal)	1.245	0.659–2.353	0.499	1.482	0.644–3.408	0.354	
SCCA normal (***vs.*** Abnormal)	1.000	0.542–1.934	0.941	0.687	0.242–1.951	0.481	
NSE normal (***vs.*** Abnormal)	1.087	0.514–2.297	0.828	0.150	0.056–0.397	**0.013**	
CA125 normal (***vs.*** Abnormal)	1.214	0.550–2.678	0.631	1.393	0.424–4.579	0.585	
PORT (***vs.*** No)	1.234	0.671–2.269	0.498	1.460	0.785–2.712	0.232	
Adjuvant chemotherapy (***vs.*** No)	0.433	0.251–0.747	**0.003**	0.459	0.244–0.862	**0.016**	

C-LCNEC, combined large-cell neuroendocrine carcinoma; C-SCLC, combined small-cell lung cancer; HR, hazard ratio; CI, confidence interval; VPI, Visceral pleural invasion; CEA, carcinoembryonic antigen; CYFRA21-1, Cytokeratin-19-fragment; SCCA, squamous cell carcinoma antigen; NSE, neuron-specific enolase; PORT, postoperative adjuvant radiotherapy

In bold: P < 0.05.

**Table 3:** Impact of clinical characteristics on overall survival in patients with resected C-LCNEC and C-SCLC by univariate analysis.

Characteristic	C-LCNEC							C-SCLC		
	HR			95% Cl	P			HR	95% Cl	P
Age ≥65 (***vs.***<65)		1.933	1.038–3.600			**0.038**		0.684	0.374–1.249	0.217
Gender male (***vs.*** Female)		1.68	1.794–3.560			0.175		1.346	0.479–3.784	0.573
Smoking history (***vs.*** No)		0.989	0.716–1.366			0.948		0.864	0.446–1.605	0.666
Tumor Size		1.287	1.046–1.583			**0.017**		1.154	1.00–1.331	**0.050**
pT stage (***vs.*** T1)										
T2		0.991	0.420–2.336			0.983		1.228	0.591–2.551	0.581
T3		3.160	1.197–8.340			0.020		2.691	1.273–5.691	**0.010**
T4		5.226	1.064–25.667			0.042		1.522	0.343–6.745	0.580
pN stage (***vs.*** N2)										
N0		0.199	0.098–0.404			**<0.001**		0.339	0.161–0.714	**0.004**
N1		0.405	0.162–1.012			0.053		1.339	0.653–2.742	0.425
Tumor location Central (***vs.*** Peripheral)		1.284	0.537–3.070			0.574		0.666	0.362–1.223	0.190
Combined components AD (***vs.*** SCC)		0.805	0.583–1.111			0.187		2.144	1.165–3.947	**0.014**
VPI (***vs.*** Without)		0.959	0.467–1.972			0.910		0.736	0.406–1.332	0.311
Primary Site (***vs.*** upper lobe)		1.327	0.967–1.823			0.080		1.126	0.827–1.534	0.449
Middle lobe		0.567	0.299–1.075			0.082		0.792	0.425–1.477	0.464
Lower lobe		1.065	0.247–4.584			0.933		1.082	0.363–3.225	0.888
Laterality		1.445	0.754–2.772			0.268		1.247	0.691–2.248	0.464
CEA normal (***vs.*** Abnormal)		0.272	0.134–0.554			**<0.001**		1.837	0.893–3.779	0.098
CYFRA21-1 normal (***vs.*** Abnormal)		1.630	0.752–3.532			0.215		1.271	0.480–3.359	0.629
SCCA normal (***vs.*** Abnormal)		1.204	0.544–2.663			0.647		1.036	0.398–2.697	0.943
NSE normal (***vs.*** Abnormal)		1.416	0.583–3.442			0.443		0.366	0.138–0.968	0.043
CA125 normal (***vs.*** Abnormal)		0.758	0.230–2.500			0.649		1.655	0.550–0.479	0.410
PORT (***vs.*** No)		1.104	0.485–2.513			0.813		1.331	0.697–2.541	0.386
Adjuvant chemotherapy (***vs.*** No)		0.382	0.198–0.737			**0.004**		0.385	0.207–0.715	**0.003**

C-LCNEC, combined large-cell neuroendocrine carcinoma; C-SCLC, combined small-cell lung cancer; HR, hazard ratio; CI, confidence interval; VPI, Visceral pleural invasion; CEA, carcinoembryonic antigen; CYFRA21-1, Cytokeratin-19-fragment; SCCA, squamous cell carcinoma antigen; NSE, neuron-specific enolase; PORT, postoperative adjuvant radiotherapy

In bold: P < 0.05.

## Discussion

Pulmonary neuroendocrine carcinoma is relatively aggressive and its diagnosis linked to worse survival in comparison with other lung cancer subtypes. Because the role of surgery has not been fully recognized before, chemotherapy combined with radiotherapy has been the major treatment for pulmonary neuroendocrine carcinoma for a long time. Pathological diagnosis through small biopsies and cytological specimens is often inaccurate and may mislead the diagnosis of C-LCNEC and C-SCLC. Based on more recent large retrospective studies, it was found that stage I SCLC could benefit from surgery with a 5-year OS of about 52% (17–19). Thus, the National Comprehensive Cancer Network (NCCN) guidelines recommend surgical treatment for patients with very limited disease (clinical T1−2, N0, M0) (20). As a subtype of NSCLC, surgery was the primary treatment for localized (I–IIIA) LCNEC. Survival benefits from surgery for patients with resectable LCNEC have also been demonstrated in several studies (21–23). Because patients could benefit more from surgery than first-line chemoradiotherapy in early-stage patients (24, 25), the number of patients received surgery has gradually increased, which may provide more available tissue for pathologists to accurately identify the combined components. To our knowledge, this study is the first, with relatively large sample size, to describe the clinicopathological features and prognosis of C-LCNEC and C-SCLC patients who had undergone surgery, and make relevant comparison between them.

According to a series of previous studies, it was reportedly to be comparable regarding clinicopathological characteristics and prognosis between LCNEC and SCLC (26, 27). However, Varlotto JM et al. analyzed the data from Surveillance, Epidemiology, and End Results Program (SEER) database and found that there were significant differences between LCNEC and SCLC in tumor size, lymph node metastasis, and differentiation degree (10). However, there was lack of evidence for clinical feature comparisons between C-LCNEC and C-SCLC. In our study we found that C-SCLC patients tended to have a history of smoking than C-LCNEC patients. C-LCNEC were mainly located at periphery lung, while C-SCLC were the tumors mainly in central location. In addition, the pathological staging of C-SCLC was more advanced compared to C-LCNEC, but C-LCNEC was characterized with VPI phenotype and combined AD components with manifestation of higher proportion than C-SCLC. These differences revealed may provide a potential tool for us to distinguish C-LCNEC from C-SCLC.

As expected, the clinical factors that influence the prognosis of C-SCLC and C-LCNEC were also revealed to be not identical. We found that tumor size, pN stage, peripheral CEA level, and adjuvant chemotherapy were independently prognostic factors for DFS and OS of C-LCNEC patients. Our study was first to analyze the prognostic factors of C-LCNEC in the presence of relatively large sample size and may make better understanding of prognosis of such a low-incidence carcinoma type. In line with previous studies, for C-SCLC patients, peripheral NSE level, pT stage, pN stage, VPI, and adjuvant chemotherapy were independently survival predictors for DFS and OS (4, 28).

Because of low incidence, survival comparison has not been previously reported between C-SCLC and C-LCNEC cancers. The previous focus was mainly on generalized SCLC and LCNEC, and demonstrated a better prognosis of LCNEC than SCLC (11, 12, 29). But other studies found no difference in prognosis between the two groups (26, 30, 31). In our study, we compared the outcomes of C-LCNEC with C-SCLC and revealed a prolonged OS of patients with C-LCNEC than those with C-SCLC, but the DFS was comparable. After PSM sampling, the prolonged OS and DFS were both statistically significant for the comparison of C-LCNEC *versus* C-SCLC, likely due to the higher neuroendocrine component of C-SCLC in nature.

It has been reported that the presence of driver mutations was very rare in LCNEC and SCLC but usually occurred in combined subtypes (32–34). Yokomizo et al. found *EGFR* mutations in three of 15 (20%) patients with C-SCLC (35). In another study, NGS was performed in 10 C-LCNEC patients, and five of them had driver gene alteration (32). Natasha et al. performed targeted next-generation sequencing testing on 45 LCNEC patients and classified LCNEC into two major types: SCLC-like (n=18; 40%), characterized byTP53+RB1 co-mutation/loss; NSCLC-like (n=25; 68%), characterized by the lack of coaltered TP53+RB1 (36). This study demonstrated the biological heterogeneity of LCNEC and provided reference for the classification and management of LCNEC patients.

In our study, *EGFR* mutations were presented in 18 patients (25.7%) and 4 patients (5.7%), for C-LCNEC and C-SCLC, respectively. *ALK* rearrangement was only observed in patients with C-LCNEC, and we did not detect alterations in other driver genes for both carcinoma types. A total of 10 patients received TKI treatment after distant metastasis and obtained good survival benefits, with an ORR rate of 60%, which suggests that genetic testing for patients with combined HGNEC is feasible and beneficial, especially for C-LCNEC patients.

Collectively, these results suggest that patients with C-LCNEC and C-SCLC may have different genetic background, which is a potential reason for the different clinical features and prognosis between them.

This article has several limitations. First of all, although we adopted the PSM method to reduce the confounders, the retrospective design of the present study may inevitably introduce bias. Second, although our sample size was relatively larger compared with previous studies, the limited sample size when grouped into C-LCNEC and C-SCLC may reduce the statistical power. Lastly, only a small number of patients in this study underwent driver gene testing, and lack of comprehensive genetic testing made it unable to capture a full spectrum of genomic profiles of HGNEC. Therefore, larger and more comprehensive studies were needed to validate and refine our findings.

In conclusion, the driver mutation landscape between C-LCNEC and C-SCLC acted in a different way, which is a potential cause for different distribution of clinicopathological features and prolonged outcome of C-LCNEC patients. Comparisons of the genetic and clinical dimensions between the two low-incidence carcinoma types may provide potential tools for clinical treatment decision.

## Data Availability Statement

The raw data supporting the conclusions of this article will be made available by the authors, without undue reservation.

## Ethics Statement

The studies involving human participants were reviewed and approved by Shanghai Chest Hospital and affiliation of ethics committee. A written informed consent was signed by included patients before they donated tumor tissues for the purpose of scientific research.

## Author Contributions

YW, FQ, and YC both have substantial contributions to the conception or design of the work, the collection and analysis of data, and the writing and editing of the article. The rest authors have given substantial contributions to the work by providing editing and writing assistance. All authors contributed to the article and approved the submitted version.

## Funding

This research was supported by the foundation of Shanghai Shenkang Hospital Development Center Emerging Frontier Technology Joint Research Project (Project No. SHDC12015115); the program of system biomedicine innovation center from Shanghai Jiao Tong University (Project No. 15ZH4009); Shanghai Jiao Tong University School of Medicine (Project No. 15ZH1008), and the foundation of Chinese Society of Clinical Oncology (Project No. Y2019AZZD-0355).

## Conflict of Interest

The authors declare that the research was conducted in the absence of any commercial or financial relationships that could be construed as a potential conflict of interest.

## Publisher’s Note

All claims expressed in this article are solely those of the authors and do not necessarily represent those of their affiliated organizations, or those of the publisher, the editors and the reviewers. Any product that may be evaluated in this article, or claim that may be made by its manufacturer, is not guaranteed or endorsed by the publisher.

## References

[1] Travis WD Brambilla E Nicholson AG Yatabe Y Austin JHM Beasley MB. The 2015 World Health Organization Classification of Lung Tumors: Impact of Genetic, Clinical and Radiologic Advances Since the 2004 Classification. J Thorac Oncol (2015) 10:1243–60. doi: 10.1097/JTO.0000000000000630 26291008

[2] Gustafsson BI Kidd M Chan A Malfertheiner MV Modlin IM. Bronchopulmonary Neuroendocrine Tumors. Cancer (2008) 113:5–21. doi: 10.1002/cncr.23542 18473355

[3] Travis WD. Pathology and Diagnosis of Neuroendocrine Tumors: Lung Neuroendocrine. Thorac Surg Clin (2014) 24:257–66. doi: 10.1016/j.thorsurg.2014.04.001 25065926

[4] Zhang C Yang H Zhao H Lang B Yu X Xiao P. Clinical Outcomes of Surgically Resected Combined Small Cell Lung Cancer: A Two-Institutional Experience. J Thorac Dis (2017) 9:151–8. doi: 10.21037/jtd.2017.01.07 PMC5303084 28203418

[5] Babakoohi S Fu P Yang M Linden PA Dowlati A. Combined SCLC Clinical and Pathologic Characteristics. Clin Lung Cancer (2013) 14:113–9. doi: 10.1016/j.cllc.2012.07.002 23010092

[6] Mangum MD Greco FA Hainsworth JD Hande KR Johnson DH. Combined Small-Cell and Non-Small-Cell Lung Cancer. J Clin Oncol (1989) 7:607–12. doi: 10.1200/JCO.1989.7.5.607 2540288

[7] Zhang JT Li Y Yan LX Zhu ZF Dong XR Chu Q. Disparity in Clinical Outcomes Between Pure and Combined Pulmonary Large-Cell Neuroendocrine Carcinoma: A Multi-Center Retrospective Study. Lung Cancer (2020) 139:118–23. doi: 10.1016/j.lungcan.2019.11.004 31775086

[8] Battafarano RJ Fernandez FG Ritter J Meyers BF Guthrie TJ Cooper JD. Large Cell Neuroendocrine Carcinoma: An Aggressive Form of Non-Small Cell Lung Cancer. J Thorac Cardiovasc Surg (2005) 130:166–72. doi: 10.1016/j.jtcvs.2005.02.064 15999058

[9] Grand B Cazes A Mordant P Foucault C Dujon A Guillevin EF. High Grade Neuroendocrine Lung Tumors: Pathological Characteristics, Surgical Management and Prognostic Implications. Lung Cancer (2013) 81:404–9. doi: 10.1016/j.lungcan.2013.05.008 23769675

[10] Varlotto JM Medford-Davis LN Recht A Flickinger JC Schaefer E Zander DS. Should Large Cell Neuroendocrine Lung Carcinoma Be Classified and Treated as a Small Cell Lung Cancer or With Other Large Cell Carcinomas? J Thorac Oncol (2011) 6:1050–8. doi: 10.1097/JTO.0b013e318217b6f8 21566535

[11] Isaka M Nakagawa K Ohde Y Okumura T Watanabe R Ito I. A Clinicopathological Study of Peripheral, Small-Sized High-Grade Neuroendocrine Tumours of the Lung: Differences Between Small-Cell Lung Carcinoma and Large-Cell Neuroendocrine Carcinoma. Eur J Cardiothorac Surg (2012) 41:841–6. doi: 10.1093/ejcts/ezr132 22219429

[12] Wang J Ye L Cai H Jin M. Comparative Study of Large Cell Neuroendocrine Carcinoma and Small Cell Lung Carcinoma in High-Grade Neuroendocrine Tumors of the Lung: A Large Population-Based Study. J Cancer (2019) 10:4226–36. doi: 10.7150/jca.33367 PMC6691699 31413741

[13] Derks JL Hendriks LE Buikhuisen WA Groen HJ Thunnissen E van Suylen RJ. Clinical Features of Large Cell Neuroendocrine Carcinoma: A Population-Based Overview. Eur Respir J (2016) 47:615–24. doi: 10.1183/13993003.00618-2015 26541538

[14] Hallet J Law CH Cukier M Saskin R Liu N Singh S. Exploring the Rising Incidence of Neuroendocrine Tumors: A Population-Based Analysis of Epidemiology, Metastatic Presentation, and Outcomes. Cancer (2015) 121:589–97. doi: 10.1002/cncr.29099 25312765

[15] Goldstraw P Chansky K Crowley J Rami-Porta R Asamura H Eberhardt WE. The IASLC Lung Cancer Staging Project: Proposals for Revision of the TNM Stage Groupings in the Forthcoming (Eighth) Edition of the TNM Classification for Lung Cancer. J Thorac Oncol (2016) 11:39–51. doi: 10.1016/j.jtho.2015.09.009 26762738

[16] Austin PC. An Introduction to Propensity Score Methods for Reducing the Effects of Confounding in Observational Studies. Multivariate Behav Res (2011) 46:399–424. doi: 10.1080/00273171.2011.568786 21818162 PMC3144483

[17] Vallieres E Shepherd FA Crowley J Van Houtte P Postmus PE Carney D. The IASLC Lung Cancer Staging Project: Proposals Regarding the Relevance of TNM in the Pathologic Staging of Small Cell Lung Cancer in the Forthcoming (Seventh) Edition of the TNM Classification for Lung Cancer. J Thorac Oncol (2009) 4:1049–59. doi: 10.1097/JTO.0b013e3181b27799 19652623

[18] Yu JB Decker RH Detterbeck FC Wilson LD. Surveillance Epidemiology and End Results Evaluation of the Role of Surgery for Stage I Small Cell Lung Cancer. J Thorac Oncol (2010) 5:215–9. doi: 10.1097/JTO.0b013e3181cd3208 20101146

[19] Badzio A Kurowski K Karnicka-Mlodkowska H Jassem J. A Retrospective Comparative Study of Surgery Followed by Chemotherapy *vs*. Non-Surgical Management in Limited-Disease Small Cell Lung Cancer. Eur J Cardiothorac Surg (2004) 26:183–8. doi: 10.1016/j.ejcts.2004.04.012 15200999

[20] Kalemkerian GP Loo BW Akerley W Attia A Bassetti M Boumber Y. NCCN Guidelines Insights: Small Cell Lung Cancer, Version 2.2018. J Natl Compr Canc Netw (2018) 16:1171–82. doi: 10.6004/jnccn.2018.0079 30323087

[21] Naidoo J Santos-Zabala ML Iyriboz T Woo KM Sima CS Fiore JJ. Large Cell Neuroendocrine Carcinoma of the Lung: Clinico-Pathologic Features, Treatment, and Outcomes. Clin Lung Cancer (2016) 17:e121–9. doi: 10.1016/j.cllc.2016.01.003 PMC5474315 26898325

[22] Fournel L Falcoz PE Alifano M Charpentier MC Boudaya MS Magdeleinat P. Surgical Management of Pulmonary Large Cell Neuroendocrine Carcinomas: A 10-Year Experience. Eur J Cardiothorac Surg (2013) 43:111–4. doi: 10.1093/ejcts/ezs174 22529187

[23] Sakurai H Asamura H. Large-Cell Neuroendocrine Carcinoma of the Lung: Surgical Management. Thorac Surg Clin (2014) 24:305–11. doi: 10.1016/j.thorsurg.2014.05.001 25065932

[24] Combs SE Hancock JG Boffa DJ Decker RH Detterbeck FC Kim AW. Bolstering the Case for Lobectomy in Stages I, II, and IIIA Small-Cell Lung Cancer Using the National Cancer Data Base. J Thorac Oncol (2015) 10:316–23. doi: 10.1097/JTO.0000000000000402 25319182

[25] Gaspar LE McNamara EJ Gay EG Putnam JB Crawford J Herbst RS. Small-Cell Lung Cancer: Prognostic Factors and Changing Treatment Over 15 Years. Clin Lung Cancer (2012) 13:115–22. doi: 10.1016/j.cllc.2011.05.008 22000695

[26] Asamura H Kameya T Matsuno Y Noguchi M Tada H Ishikawa Y. Neuroendocrine Neoplasms of the Lung: A Prognostic Spectrum. J Clin Oncol (2006) 24:70–6. doi: 10.1200/JCO.2005.04.1202 16382115

[27] Iyoda A Hiroshima K Baba M Saitoh Y Ohwada H Fujisawa T. Pulmonary Large Cell Carcinomas With Neuroendocrine Features Are High-Grade Neuroendocrine Tumors. Ann Thorac Surg (2002) 73:1049–54. doi: 10.1016/S0003-4975(01)03616-5 11996239

[28] Lei Y Feng H Qiang H Shang Z Chang Q Qian J. Clinical Characteristics and Prognostic Factors of Surgically Resected Combined Small Cell Lung Cancer: A Retrospective Study. Lung Cancer (2020) 146:244–51. doi: 10.1016/j.lungcan.2020.06.021 32592985

[29] Kinslow CJ May MS Saqi A Shu CA Chaudhary KR Wang TJC. Large-Cell Neuroendocrine Carcinoma of the Lung: A Population-Based Study. Clin Lung Cancer (2020) 21:e99–113. doi: 10.1016/j.cllc.2019.07.011 31601526

[30] Mochizuki E Matsuura S Oishi K Miyashita K Ichijyo K Furukawa S. Surgical Resection for Clinical Stage I High-Grade Neuroendocrine Carcinoma of the Lung. World J Surg Oncol (2018) 16:33. doi: 10.1186/s12957-018-1337-2 29454358 PMC5816519

[31] Kinoshita T Yoshida J Ishii G Aokage K Hishida T Nagai K. The Differences of Biological Behavior Based on the Clinicopathological Data Between Resectable Large-Cell Neuroendocrine Carcinoma and Small-Cell Lung Carcinoma. Clin Lung Cancer (2013) 14:535–40. doi: 10.1016/j.cllc.2013.04.003 23792008

[32] Miyoshi T Umemura S Matsumura Y Mimaki S Tada S Makinoshima H. Genomic Profiling of Large-Cell Neuroendocrine Carcinoma of the Lung. Clin Cancer Res (2017) 23:757–65. doi: 10.1158/1078-0432.CCR-16-0355 27507618

[33] Lou G Yu X Song Z. Molecular Profiling and Survival of Completely Resected Primary Pulmonary Neuroendocrine Carcinoma. Clin Lung Cancer (2017) 18:e197–201. doi: 10.1016/j.cllc.2016.11.014 28024928

[34] Gugger M Burckhardt E Kappeler A Hirsiger H Laissue JA Mazzucchelli L. Quantitative Expansion of Structural Genomic Alterations in the Spectrum of Neuroendocrine Lung Carcinomas. J Pathol (2002) 196:408–15. doi: 10.1002/path.1065 11920736

[35] Yokomizo A Tindall DJ Drabkin H Gemmill R Franklin W Yang P. PTEN/MMAC1 Mutations Identified in Small Cell, But Not in Non-Small Cell Lung Cancers. Oncogene (1998) 17:475–9. doi: 10.1038/sj.onc.1201956 9696041

[36] Rekhtman N Pietanza MC Hellmann MD Naidoo J Arora A Won H. Next-Generation Sequencing of Pulmonary Large Cell Neuroendocrine Carcinoma Reveals Small Cell Carcinoma-Like and Non-Small Cell Carcinoma-Like Subsets. Clin Cancer Res (2016) 22:3618–29. doi: 10.1158/1078-0432.CCR-15-2946 PMC4995776 26960398

