# Supplementary material for: Comparative Study of Pulmonary Combined Large-Cell Neuroendocrine Carcinoma and Combined Small-Cell Carcinoma in Surgically Resected High-Grade Neuroendocrine Tumors of the Lung
Source: Front Oncol. 2021 Sep 22;11:714549. doi: 10.3389/fonc.2021.714549 (PMC8493068; doi:10.3389/fonc.2021.714549)
Supplement: Supplementary file 1 [file Table_1.docx]

**Supplemental Table1 Details of C-LCNEC and C-SCLC patients receiving TKI therapy.**

| **No.** | **Histology** | **Gender** | **Age** | **Smoking** | **Metastasis Status** | **Alterations** | **TKI Therapy** | **PFS** | **Best Response** |
| --- | --- | --- | --- | --- | --- | --- | --- | --- | --- |
| **1** | C-LCNEC/AD | Female | 37 | Never | Bone | EML4-ALK | Crizotinib | 6 months | SD |
| **2** | C-LCNEC/AD | Male | 63 | Smoker | Bone | EML4-ALK | Crizotinib | 11 months | PR |
| **3** | C-LCNEC/AD | Male | 77 | Smoker | Brain | EML4-ALK | Crizotinib | not reached | PR |
| **4** | C-LCNEC/AD | Male | 46 | Smoker | Lung | EML4-ALK | Crizotinib | not reached | PR |
| **5** | C-LCNEC/AD | Male | 62 | Never | Lung | EGFR 19del | Elotinib | not reached | SD |
| **6** | C-LCNEC/AD | Male | 51 | Never | Bone | EGFR 19del | Gefitinib | not reached | PR |
| **7** | C-LCNEC/AD | Male | 45 | Never | Brain | EGFR 19del | Gefitinib | 10 months | SD |
| **8** | C-LCNEC/AD | Female | 50 | Never | Lung | EGFR 21L858R | Afatinib | 7 months | PR |
| **9** | C-LCNEC/AD | Female | 56 | Never | Brain | EGFR 19del | Icotinib | not reached | PR |
| **10** | C-SCLC/AD | Female | 58 | Never | Lung | EGFR 19del | Icotinib | 5 months | SD |
